# Fabrication and Evaluation of Dissolving Hyaluronic Acid Microneedle Patches for Minimally Invasive Transdermal Drug Delivery by Nanoimprinting

**DOI:** 10.3390/gels11020089

**Published:** 2025-01-23

**Authors:** Sayaka Miura, Rio Yamagishi, Mano Ando, Yuna Hachikubo, Nor Amirrah Ibrahim, Nur Izzah Md Fadilah, Manira Maarof, Misaki Oshima, Sen Lean Goo, Hiryu Hayashi, Mayu Morita, Mh Busra Fauzi, Satoshi Takei

**Affiliations:** 1 Department of Pharmaceutical Engineering, Toyama Prefectural University, Imizu 939-0398, Toyama, Japan; sayaka13579@outlook.jp (S.M.); rio.yamagishi@outlook.jp (R.Y.); mano_ando@outlook.jp (M.A.); y.hachikubo@outlook.com (Y.H.); misaki_oshima@outlook.jp (M.O.); senlean.goo@outlook.com (S.L.G.); hiryu0821@outlook.jp (H.H.); mayu_.morita@outlook.jp (M.M.); 2 Department of Tissue Engineering and Regenerative Medicine, Faculty of Medicine, Universiti Kebangsaan Malaysia, Kuala Lumpur 56000, Malaysia; noramirrahibrahim@gmail.com (N.A.I.); izzahfadilah@ukm.edu.my (N.I.M.F.); manira@ukm.edu.my (M.M.); fauzibusra@ukm.edu.my (M.B.F.); 3 Advance Bioactive Materials-Cells UKM Research Group, University Kebangsaan Malaysia, Bangi 43600, Malaysia

**Keywords:** microneedle patch, micro/nano technology, drug delivery, hyaluronic acid soft gel, skin penetration, drug permeability, minimally invasive, biomedical applications, gas-permeable MN mold

## Abstract

Transdermal drug delivery minimizes pain and provides a controlled, stable release of drugs, but its effectiveness is limited by the skin’s natural barriers. Microneedles overcome this problem, enabling minimally invasive drug delivery. Microneedle patches (MNPs) with 80 µm-tall needles composed of hyaluronic acid (HA) were developed and evaluated for their formability, structural integrity, dissolution rate, skin penetration ability, and drug transmission capacity. The influence of the molecular weight of HA on these properties was also investigated. MNPs made from low-molecular-weight HA (30 kDa–50 kDa) demonstrated 12.5 times superior drug permeability in ex vivo human skin compared to needleless patches (NLPs). Furthermore, in the same test, low-molecular-weight HA MNPs had 1.7 times higher drug permeability than high-molecular-weight HA MNPs, suggesting superior transdermal administration. The molecular weight of HA significantly influenced its solubility and permeability, highlighting the potential effectiveness of MNPs as drug delivery systems. Puncture tests demonstrated a penetration depth of 50–60 µm, indicating minimal nerve irritation in the dermis and effective drug delivery to the superficial dermal layer. These results present a manufacturing technique for MNPs incorporating model drug compounds and highlight their potential as a novel and minimally invasive drug delivery method for the biomedical applications of soft gels.

## 1. Introduction

The efficacy of a drug is contingent upon not only the active pharmaceutical ingredients, but also the method of administration [1,2]. Although oral administration is considered convenient and can be administered without causing discomfort to patients [3], it may be less effective due to first-pass metabolism and the lack of stability of drug concentration in the blood [4], and may not be suitable for hydrophobic drugs [5]. Injections exhibit high bioavailability and the rapid onset of drug effects [6]; however, they require medical personnel for administration and are associated with pain, leading to low patient compliance [7]. Furthermore, needle phobia presents an additional concern, potentially causing a variety of physical, psychological, and behavioral symptoms [8].

Transdermal drug delivery, which administers drugs through the skin [9], offers the advantage of reducing pain and enabling a stable release of drugs compared to traditional oral and injection administration [10]. Drugs delivered transdermally circumvent digestive degradation and first-pass metabolism, resulting in high bioavailability [11,12,13]. However, the stratum corneum, the barrier function of the skin [14,15,16], permits only non-polar, hydrophobic molecules of less than 500 Da to pass through, thus limiting the delivery of larger molecules [17,18].

Microneedles (MNs) are designed to physically bypass this barrier, which is purported to enhance drug permeability [19,20]. MNs, comprising a needle array and base, are minimally invasive and elicit minimal pain and discomfort [21,22,23]. Dissolvable MNs fabricated from biocompatible synthetic polymers such as hyaluronic acid (HA) [24,25,26], polylactic acid [27], polyglycolic acid [28], and polyvinyl alcohol [29,30,31] penetrate the stratum corneum upon insertion and subsequently dissolve [32], releasing the encapsulated drug without generating harmful sharp waste or risking bloodstream infections [33,34]. Advancements in MN technology have presented new possibilities for transdermal drug delivery and expanded its applications in the medical and cosmetic fields [35].

Since the 1980s, micro/nanofabrication techniques using various polymers have attracted considerable attention [36], and the resulting micro/nanostructures have been applied in fields such as microfluidics [37], biosensors [38], microelectronics [39], and tissue engineering [40,41]. In conventional photolithography, the basis of micro/nanofabrication technology [42], structures are fabricated by exposing a photosensitive resist through a mask [43]. For example, Ikake et al. [44] developed a high-strength and tough PMMA/titania hybrid material, using sol–gel and photolithography techniques to improve material properties by molding the macropolymer.

Nanoimprint lithography (NIL) has emerged as a rapid, scalable, and cost-effective alternative to photolithography, achieving a resolution of a few nanometers [45]. This innovative technique has garnered significant attention in the semiconductor industry because of its potential to overcome the limitations of traditional lithography methods. NIL’s capacity to produce high-resolution patterns with remarkable precision opens up new possibilities for the fabrication of advanced electronic devices and nanostructures. This process utilizes a mold to replicate a pattern in a heat- or UV-sensitive resin [46]. For instance, Micheal et al. [47] employed NIL to pattern a hybrid nanoparticle/polymer composite resist into a lattice structure, which they subsequently stacked to create a 3D photonic crystal, thereby advancing the direct 3D patterning technique.

3D printing has gained attention as a method for the rapid and precise manufacturing of complex designs [48]. This is particularly valuable in the manufacturing of medical devices, enabling customized products tailored to the needs of individual patients [49]. Conventional micro/nanofabrication techniques such as lithography [50], etching [51], and injection molding [52,53] are limited by their labor-intensive processes, complexity, and high costs [54]. However, 3D printing technology, which allows for material and design tailoring, is being developed for medical materials, such as MNs.

To fabricate dissolving MNs using micromolding techniques, it is typically necessary to apply centrifugation or vacuum to remove air bubbles [55] and completely fill the tip of the mold [56]. However, centrifugation is time-consuming and difficult to control, and vacuum techniques are only effective for low-viscosity and low-concentration polymer solutions [57]. Previously, we utilized gas-permeable porous molds and NIL to fabricate MNs with heights of 20–80 µm without centrifugation or vacuum [58,59], achieving significantly smaller structures than conventional MNs with heights of 100–1000 µm. This reduction in size is expected to further minimize pain and discomfort; however, few studies have focused on such small MNs, and it is essential to evaluate their effectiveness in practical applications. In this study, we accurately fabricated MN patches (MNP) with a height of 80 µm using NIL and evaluated the mechanical properties, dissolubility, and biological properties of the MNP. We fabricated MNPs made from HA with two different molecular weights, and considered the differences between them. Many other MN studies have evaluated MNs using animal skin [60,61,62], such as rats and pigs, which are known to be biologically different from the human skin [63]. We used excised human skin to observe the skin section in the MNP puncture test and to examine the permeation profile in the ex vivo permeation test.

By obtaining highly accurate data that more precisely reflects the effects during clinical use, this study will demonstrate the effectiveness of MNPs made with HA in biomedical applications and contribute to research on advanced soft gels.

## 2. Results and Discussion

### 2.1. Microneedle Shape Observation Results

The primary challenge in fabricating dissolving MNPs with a height of 80 µm from a high-viscosity HA solution is the formation of an MN structure without defects. This study presents a method to address this challenge in an ambient-pressure environment utilizing gas-permeable MN molds instead of conventional centrifugal or vacuum methods. Gas-permeable MN molds possess a porous structure and demonstrate permeability to oxygen and carbon dioxide [64], facilitating the accurate molding of MN despite the high viscosity of HA solutions due to their capacity to fill the molds. The viscosity of the HA solution constituting each MNP in the environment (25 °C) when the gas-permeable MN mold was pressed into the HA solution was 400 Pa·s for MNP_40k and 390 Pa·s for MNP_80k.

**Figure 1:**
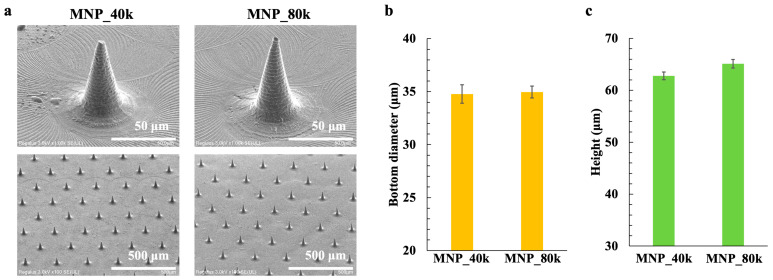
MN shape observations and dimensions. (**a**) SEM images of MNP_40k and MNP_80k, (**b**) needle bottom diameter, and (**c**) needle height.

Figure 1a depicts the scanning electron microscope (SEM) images from the transfer of MNPs of two different HA molecular weights. Henceforth, MNPs made of HA with molecular weights of 30 kDa–50 kDa are referred to as MNP_40k, and those with molecular weights of 50 kDa–110 kDa are referred to as MNP_80k. Gas-permeable MN molds were transferred from the master mold, and each MNP was fabricated from the gas permeable MN molds. The MNs formed on the polylactic acid extended to the tip of the needle and were transferred distinctly for both MNP_40k and MNP_80k. The spiral pattern on the surface of the needle, which was formed during the creation of the master mold by drilling, was also accurately transferred.

The needle tip length, bottom diameter, and height of the MN are important parameters for tissue penetration as they determine whether the needle can reach the target location (penetration depth) [65,66]. The tip length, bottom diameter, and height of MNP_40k and MNP_80k were statistically analyzed from the SEM images. Regarding height, SEM images are susceptible to errors from a working distance, so the values measured were converted based on the master mold height of 80 µm, which was 4.7 µm, 37 µm, and 65 µm for MNP_40k, and 3.7 µm, 36 µm, and 68 µm for MNP_80k, respectively. The master mold held values of 3.4 µm, 40 µm, and 80 µm. Figure 1b and c illustrates the bottom diameters and the heights of MNP_40k and MNP_80k. In terms of the height, each MN exhibited a deviation of less than 15 µm from the master mold. This indicates that the master mold must be designed with an error margin of approximately 15 µm when using MNs for pharmaceutical applications. Furthermore, when comparing the heights of MNP_40k and MNP_80k, MNP_80k is taller, suggesting that it has better formability. The reason for the low needle height of MNP_40k is that low-molecular-weight HA has a lower water retention capacity than high-molecular-weight HA. When it was dried at a low temperature of 5 °C during molding, the needle shape of MNP_40k was thought to shrink, owing to its low water retention capacity. In addition, MNP_80k has a higher molecular weight than MNP_40k; therefore, it is thought that the HA chains are more likely to crosslink, and that MNP_80k has better formability than MNP_40k, which has a lower molecular weight. In addition, in the viscosity measurement of the HA solution, the viscosity of the HA solution used for MNP_40k was slightly higher than that of MNP_80k. Therefore, it is thought that the pressure applied to the gas-permeable MN mold when preparing MNP_40k is insufficient for the viscosity of the HA solution. This may be improved by applying a higher pressure or by improving the molecular design of the gas-permeable MN mold to control gas permeability.

The gas-permeable MN mold employed in this study was capable of transferring MN from a high-viscosity HA solution without significant errors, even when different molecular weights of the HA solution were utilized.

A previous study by Jeong et al. [67] attempted to fabricate MNs with heights ranging from 100 µm to 1000 µm using 3D printing, but reported that MNs with a height of 100 µm could not be produced due to their extremely small size. The ability to fabricate MNs with heights below 100 µm using nanoimprinting with gas-permeable molds is considered highly advantageous.

### 2.2. Mechanical Property Test Results

**Figure 2:**
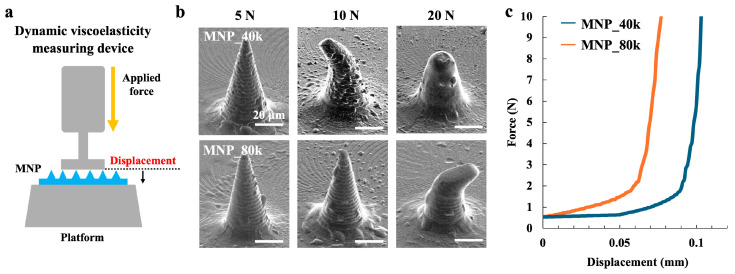
(**a**) Schematic of mechanical property tests of MNPs performed using dynamic viscoelasticity measuring device. (**b**) SEM image of MNPs after testing. Scale bar: 20 µm. (**c**) Force (N) vs. displacement (mm) curves during testing of two types of MNPs attached to the platform.

For effective and safe clinical use, MNPs must be inserted into the skin without bending or breaking it. Therefore, ensuring sufficient mechanical strength is critical for achieving transdermal drug delivery. To measure the mechanical strength of the fabricated MNPs, a dynamic viscoelasticity measuring device was used to perform mechanical property tests under vertical loading, as illustrated in Figure 2a. Figure 2b presents the SEM images of MNPs after compression at different pressures for the two types of MNPs. MNP_40k exhibited a tip deformation at 10 N and reduction at 20 N. MNP_80k exhibited minimal deformation at 10 N and tip deformation at 20 N. Notably, neither MNP type experienced needle breakage.

Figure 2c shows the force-displacement curves of the needles. When the compressive force of the MNs was 5 N (at which point neither MN was deformed), the compressive force increased from 0.5 N to 5 N as the displacement of MNP_80k increased from 0 mm to 0.07 mm. For MNP_40k, at the same displacement (0.07 mm), the compressive force was as low as 1 N. This indicates that the mechanical strength of MNP_80k in the vertical direction is superior to that of MNP_40k. Consequently, it can be inferred that the compressive force on the MN increases with increasing HA molecular weight.

It was shown that the molecular weight of the material plays an important role in the mechanical strength of MNPs. In particular, the results suggest that the use of high-molecular-weight materials should be designed to ensure puncture performance in areas of hard skin. The materials with high molecular weight have stronger intermolecular interactions and form a more rigid mechanical structure [68], which may enable effective puncture performance even at hard sites. On the other hand, the materials with low molecular weight are more flexible and have better solubility, but may not be suitable for hard skin sites because of their reduced mechanical strength. These findings indicate that the molecular weight of the material used is an important parameter to optimize in the design of MNPs.

Some studies have developed hybrid composites by cross-linking HA solutions of different molecular weights with the expectation of a synergistic effect to obtain the advantages of both [69]. In our previous study [59], MNs composed of HA with two different molecular weights demonstrated the same mechanical strength and improved solubility as MNs prepared using high-molecular-weight HA alone, thus confirming the synergistic effect of using HA with different molecular weights.

In a previous study, Makvandi et al. [70] reported that conical MNs exhibited superior mechanical strength compared to pyramidal MNs, indicating that the shape and aspect ratio of MNs are also closely related to mechanical strength. It is essential to optimize the balance between mechanical strength and solubility when developing MNPs that can be applied to areas with hard skin or a thick stratum corneum.

### 2.3. Solubility Test in Conditions That Mimicked the Skin Environment Results

**Figure 3:**
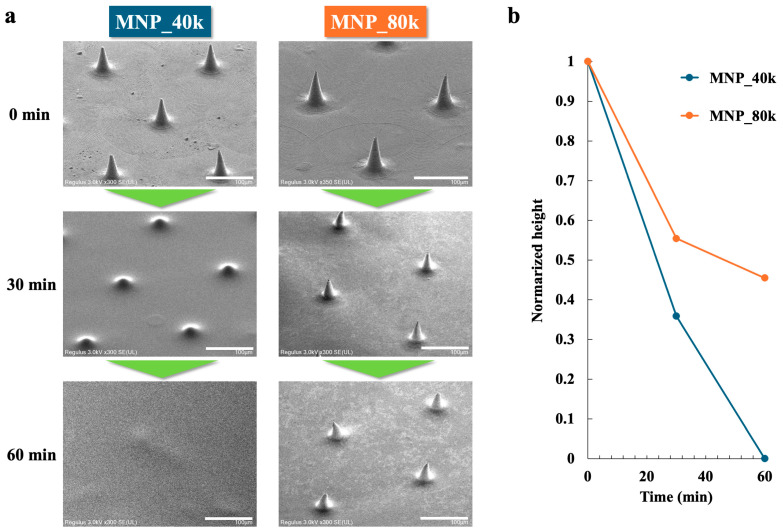
Solubility test in conditions that mimicked the skin environment (temperature 32 °C, humidity 75 ± 5%). (**a**) SEM images of MNPs at 0, 30, and 60 min after the start of the test. Scale bar: 100 µm. (**b**) Molecular weight dependence of the solubility at different times.

To evaluate the dissolution behavior in the skin, MNPs were monitored over various time periods under conditions that mimicked the skin environment. The solubility was evaluated based on the initial (0 min) height of the MNPs produced. Figure 3a presents the SEM results for MNP_40k and MNP_80k at each time point. For MNP_40k, 64% dissolved at 30 min, and complete dissolution occurred at 60 min. For MNP_80k, 45% dissolved at 30 min, incomplete dissolution occurred at 60 min, and 55% dissolved at 60 min. It is hypothesized that MNPs hydrate in the skin, form a gel, and subsequently dissolve [71]. It is probable that the MNPs in this study were dissolved via a similar process.

MNPs with different molecular weights of HA exhibit distinct dissolution behaviors in a simulated skin environment. Figure 3b illustrates the molecular weight dependence of the solubility test over time, with the MNP height at 0 min normalized to one. MNP_40k, which has a lower molecular weight, dissolves more rapidly than MNP_80k. Conversely, MNP_80k showed gradual dissolution. This observation can be attributed to higher molecular weight HA, which forms a more robust physical structure [72], thereby limiting its interaction with water. Consequently, the ability to modulate solubility based on the molecular weight of HA has significant implications for the design of MNPs drug delivery systems. This finding indicates that solubility can be regulated by adjusting the molecular weight.

### 2.4. Puncture Test Results

**Figure 4:**
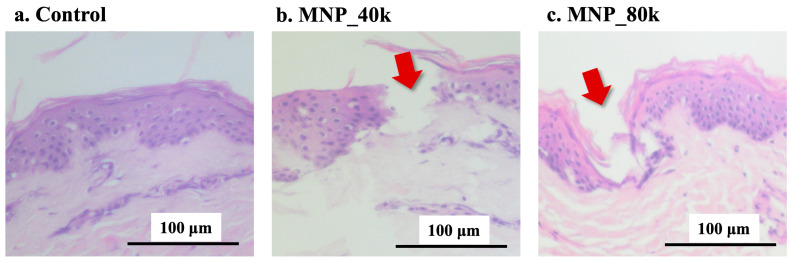
Biological evaluation of excised human skin. Images of hematoxylin and eosin (H&E)-stained (**a**) control, (**b**) MNP_40k (30 s) and (**c**) MNP_80k (30 min). Red arrows: needle marks.

Figure 4 presents optical microscopy images of skin cross-sections following MNP removal, illustrating the results of the puncture tests conducted on excised human skin. Figure 4a depicts a cross-section of the control skin, whereas Figure 4b shows a cross-section of the skin punctured with MNP_40k for 30 s. Hematoxylin and eosin (H&E) staining confirmed that the MNs penetrated the stratum corneum and perforated the dermal layer. The penetration depth is approximately 50 µm. Figure 4c illustrates a cross-section of MNP_80k punctured in the skin for 30 min. While 30 s of MNP_80k puncture was insufficient to penetrate the epidermal layer, 30 min of puncture confirmed that the MNs had penetrated the stratum corneum and perforated the dermal layer. The penetration depth was approximately 59 µm. Although the mechanical strength of MNP_80k was high, it could not be fully pierced by puncturing for 30 s. Chi et al. [73] mention that if the mechanical strength of MNs is too high, they become brittle and some of the MNs tend to break easily when the applied force increases. Owing to its high mechanical strength, MNP_80k may have become bent, as shown in Figure 2b, after puncturing for 30 s. However, during puncturing for 30 min, the bent needle at the tip may have penetrated over time and the puncture mark may have been confirmed. The penetration depth was 50 µm for MNP_40k and 59 µm for MNP_80k; however, this difference is thought to be due to the difference in puncturing time or the difference in the mechanical properties of the MNP. To consider this in more detail, additional experiments under different puncturing conditions are necessary.

The MNs developed in this study achieved a penetration depth of 50–60 µm, despite being significantly shorter in height compared to the conventional MNs typically studied [74]. One of the factors behind this is thought to be the 200 µm spacing between the needles. Supporting this hypothesis, Kochhar et al. [75] demonstrated that increasing the spacing between needles (ranging from twice to six times the base diameter) enhances the force applied per needle, thereby increasing the penetration depth into the skin. Another possible factor is the very small tip diameter of the MN. In fact, a study by Sabri et al. [76] reported that the smaller contact area between the MN and the skin increased the tip pressure, improving the penetration of the MN.

The height of the MNs should be carefully designed to enhance the effectiveness of drug delivery while minimizing any pain stimulation for the patient [71]. The MNPs utilized in this study could potentially be employed for drug delivery from the surface of the skin beyond the stratum corneum and epidermis, and localized in the superficial dermal layer (50–60 µm) [77,78]. This range was designed to avoid areas where dermal nerves are present, suggesting that drug delivery can be accomplished with minimal or no pain.

This outcome indicates the potential for a substantial improvement in patient comfort compared with conventional injections of MNs with a height of 100–1000 µm that penetrate deeper. The design of MNs to avoid the dermal layer, where the dermal nerves are located, shows promise for improving patient compliance, particularly in treatments requiring repeated application (e.g., management of chronic diseases or vaccination). Furthermore, this shallow layer-specific drug delivery may be particularly advantageous in preventing drugs from directly reaching the bloodstream and delivering localized effects. This design could be applied as a novel therapeutic strategy for the treatment of skin-related diseases and localized inflammation.

### 2.5. Ex Vivo Permeation Test Results

Ex vivo permeation tests were conducted to investigate the permeation profile of model drug compounds in MNPs punctured into excised human skin. The Franz diffusion cell utilized in this study simulates the body’s natural blood circulation using receptor and donor compartments. The skin permeation of fluorescein isothiocyanate dextran (FD4), with a molecular weight of 4 kDa employed as a model drug compound, was measured to assess drug release from MNP-treated skin samples into the receptor compartment. HA has properties such as biocompatibility, non-immunogenicity, and biodegradability [79], and is sometimes used as an active ingredient; however, in this study, HA was used as the matrix in the MNPs, and FD4 was used as a model drug compound.

**Figure 5:**
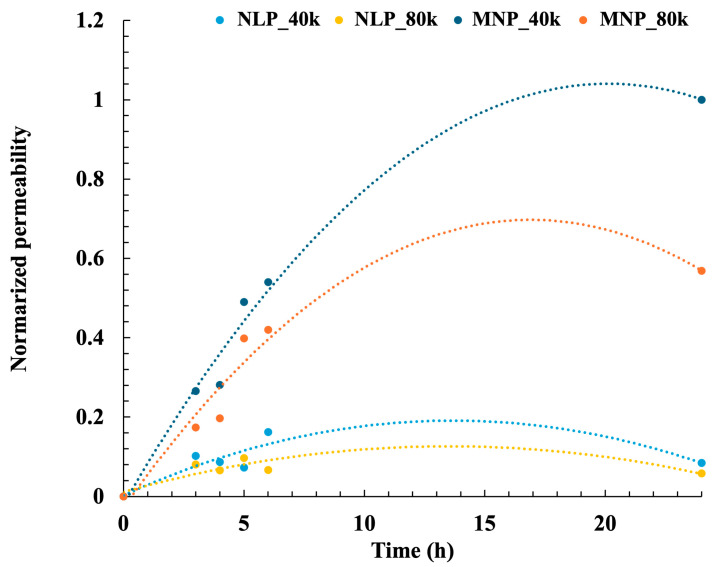
Ex vivo permeation profile of model drug compounds through the skin when delivered using NLPs and MNPs.

MNP permeation in the model drug compounds is shown in Figure 5. The permeability of the MNPs was compared to that of the needleless patch (NLP) as the control intervention. Using the permeability of MNP_40k, which demonstrated the highest permeability, as a baseline after 24 h, the result indicates strong drug permeability with MNP in comparison to NLP. Moreover, the notably high permeability of MNP_40k can be attributed to its utilization of HA with a relatively low molecular weight of 30 kDa–50 kDa, which enhances its solubility in the skin (Section 2.3) and facilitates efficient drug delivery. The permeability of MNP_40k exhibited a distinct trend 3–6 h after the initiation of the test and continued to increase for up to 24 h. Conversely, the permeability of NLP_40k was markedly low and no significant changes were observed even after 24 h.

The permeability of MNP_80k after 24 h was approximately 60% that of MNP_40k. Permeability enhancement requires additional investigation, such as extending the puncture time from 30 to 60 min. Nevertheless, MNP_80k demonstrated superior permeability compared with NLP_80k, validating MNP’s efficacy in this study. These findings indicate that the drug permeability of MNPs depends on the molecular weight of HA. In a previous study, Lahiji et al. [80] reported that drug (insulin) permeability reached 92% for MNPs at a puncture depth of 50 µm in a study using pig cadaver skin, which was significantly higher than the 25% for NLPs. Comparably, the puncture depth of 50–60 µm attained by MNP in this study supports its higher efficacy than the NLP.

It is a challenge to directly compare the skin permeability achieved from MNs of this study with other researchers because of the substantially varied test methods and conditions. However, the very small height of the MNs in this study is thought to have a slower permeation speed compared to conventional MNs [81,82] of 500 µm or more. Therefore, the MNs fabricated in this study may be effective for designing drugs with a slower permeation speed into the bloodstream. In addition, the oral mucosa has a rich blood supply, which is thought to promote successful drug penetration, and is expected to grow as an oral MN drug delivery system in the future [83]. The possibility of drug administration to the oral mucosa by taking advantage of the fineness of our MNP is also suggested. Preclinical in vivo testing would be beneficial for future research.

In conclusion, the results suggest that the MNPs in this study promoted the greater permeability of model drug compounds with a molecular weight of 4 kDa to the skin.

## 3. Conclusions

This investigation entailed the development of a microneedle patch (MNP) featuring 80 µm tall needles, which are smaller than conventional designs. Various properties of hyaluronic acid (HA)-based MNPs were evaluated, including their formability, structural integrity, dissolution rate, skin penetration ability, and drug transmission capacity. The influence of the molecular weight of HA on these characteristics was also examined. The findings demonstrated that MNPs composed of low-molecular-weight HA (30 kDa–50 kDa) exhibited 12.5 times superior drug permeability in ex vivo human skin samples compared to needleless patches (NLPs). These results highlight the significant impact of the molecular weight of HA on solubility and permeability, indicating the potential efficacy of the developed MNPs as drug delivery systems. Furthermore, the observed penetration depth (50–60 µm) during the MNP puncture tests suggests minimal nerve irritation in the dermis and efficient drug delivery to the superficial dermal layer.

The MNPs developed in this study, characterized by high solubility and drug permeability, demonstrated potential as a novel, minimally invasive, and efficacious drug delivery method. In the near future, we will focus on producing drug-loaded MNPs to elucidate their pharmacological effects and develop a manufacturing technology for MNPs with a high aspect ratio, with a view to application to areas of thicker skin. By modifying parameters such as the molecular weight of the MNP components, it is feasible to create patches suitable for vaccine administration or topical drug delivery, potentially advancing clinical applications in these domains.

## 4. Materials and Methods

The materials of the gas-permeable MN mold and MNPs used in the experiments are summarized in Supplementary Materials Table S1.

### 4.1. Methods for Fabrication of Gas-Permeable MN Molds

The following methods were employed to fabricate gas-permeable MN molds that exhibited gas permeability and could fill the needle apex. The drilled micromachined metal master mold comprised conical MNs, 80 µm in height and 40 µm in bottom diameter at the base, arranged in a 2.5 × 2.5 cm^2^ area. A 200 µm center-to-center pitch between the MNs was utilized. The UV-curable sol–gel material used to produce the gas-permeable MN molds was prepared according to previous studies [59,64,84]. The sol–gel polymerization used has the advantages of low processing temperatures [85,86,87], high homogeneity [88,89], and the ability to produce materials in a variety of forms, including coatings and thin films [90,91].

A sufficient quantity of UV-curable sol–gel material was deposited on a metal master mold that had undergone mold release treatment (DURASURF DS-831TH, Harves, Saitama, Japan), covered with a glass substrate, and subjected to a UV light source (Lightning Cure LC8, Hamamatsu Photonics, Shizuoka, Japan) for 2 min. Subsequently, the master mold was removed and carefully separated from the glass substrate to yield the film-like gas-permeable MN mold.

### 4.2. MNP Fabrication Methods

**Figure 6:**
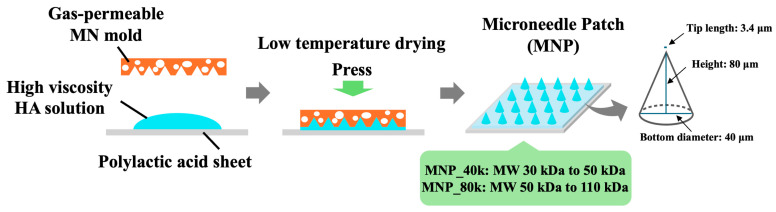
Fabrication method of HA MNP using a gas-permeable MN mold and an overview of needle shape.

Figure 6 illustrates the fabrication process of MNPs. MNPs were synthesized using two types of HA with distinct molecular weights. HA with molecular weights ranging from 30 kDa to 50 kDa (SANCT) and HA with molecular weights ranging from 50 kDa to 110 kDa (FCH-SU, Kikkoman Biochemifa, Tokyo, Japan) were utilized. Water was added so that the concentrations were 26.3 wt% for HA with molecular weights of 30 kDa–50 kDa and 14.6 wt% for HA with molecular weights of 50 kDa–110 kDa. Dynamic viscoelasticity measurements of the HA solution were performed using a dynamic viscoelasticity measuring device (MCR102, Anton Paar Japan, Tokyo, Japan). The viscosity of the HA solution was measured in oscillatory rotation mode (parallel plate diameter; 50 mm, gap; 0.5 mm, shear rate; 0.01 s^−1^, temperature; 25 °C). The HA solution was solubilized using an ultrasonic washing apparatus (OZL-2000, Onezili, Guangzhou, China). Subsequently, the high-viscosity HA solution was transferred to an Eppendorf tube and subjected to defoaming in a centrifuge (CF0201001, FOUR E’s Scientific, Guangzhou, China). The high-viscosity HA solution was then deposited on a polylactic acid sheet (Dai-ichi Kogyo Seiyaku, Kyoto, Japan) that had undergone ozone treatment for 5 min using UV ozone treatment equipment (LT0Z-180, Litho Tech Japan, Saitama, Japan), followed by the placement of a gas-permeable MN mold on top. The mold was subjected to pressure and allowed to desiccate for 5 or 7 days under refrigerated conditions. MNPs were obtained by removing the mold. They were designated as MNP_40k (HA with molecular weights of 30 kDa–50 kDa) and MNP_80k (HA with molecular weights of 50 kDa–110 kDa), respectively. The morphology of the MNPs was examined using cold-field emission SEM (Regulus8100, Hitachi High-Tech, Tokyo, Japan).

### 4.3. Mechanical Property Test

The mechanical properties of the MNPs were measured using a dynamic viscoelasticity measuring device (MCR102, Anton Paar Japan, Tokyo, Japan). In the test, MNPs of 2.5 × 2.5 cm^2^ in size were secured on the platform surface of the dynamic viscoelasticity measuring device. The measuring fixture (12 mm in diameter) was subsequently moved vertically at a constant speed of 1 µm/s to compress the MNPs. Compression was terminated when the force reached 5, 10, or 20 N, and the morphology of each MNP was observed by SEM. The displacement was set to 0 mm when the bottom of the measuring fixture contacted the tip of the MN and a compression force of 0.5 N was reached. The force-displacement curve of the needle was obtained by recording the displacement of the sensor and the force applied to the MNP until the compression force reached 10 N.

### 4.4. Solubility Test in Conditions That Mimicked the Skin Environment

The MNP solubility test, which simulates the skin environment, was conducted as follows. MNP_40k and MNP_80k were placed in an incubator (PIC-101, AZ ONE, Osaka, Japan) at a temperature of 32 °C and humidity of 75 ± 5%. After 30 min or 60 min, the MNPs were removed and examined using a cold-field emission SEM.

### 4.5. Skin Sampling

The experimental protocol using excised human skin was approved by the Universiti Kebangsaan Malaysia Research Ethics Committee (UKM PPI/111/8/JEP-2024-906). The redundant skin samples were obtained from consenting patients undergoing abdominal surgery at the Hospital Canselor Tuanku Mukhriz (HCTM). As much subcutaneous fat was removed from the skin as possible, stored in a freezer, and thawed in a refrigerator before use.

### 4.6. Puncture Test

Puncture levels were evaluated in the excised human skin after MNP puncture. The skin sample was placed on parafilm and secured on all four sides with a needle. MNPs were punctured into the skin sample for 30 s, followed by tissue processing. Alternatively, the MNPs were punctured for 30 s and left in situ on the skin for 30 min, followed by tissue processing. Tissue processing included fixation, dehydration, clearing, paraffin embedding, sectioning, and H&E staining. Skin samples were examined under a binocular microscope (CH30, Olympus, Tokyo, Japan).

### 4.7. Ex Vivo Permeation Test

**Figure 7:**
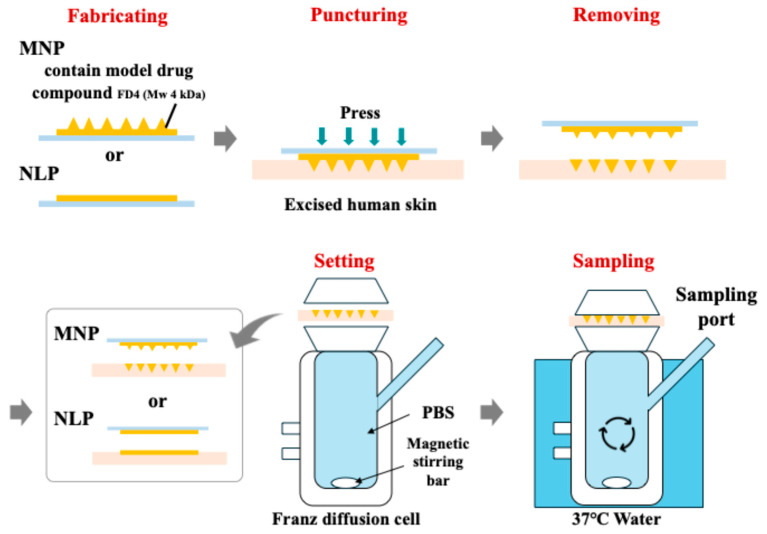
Methods of ex vivo permeation tests for evaluating the permeation behavior of MNPs using excised human skin.

Figure 7 illustrates the methods of ex vivo permeation tests. Ex vivo permeation tests were conducted to examine the permeation behavior of model drug compounds through MNP-treated excised human skin. To each of the two high-viscosity HA solutions of different molecular weights described previously, 20 mg/mL FD4 (Fluorescein isothiocyanate-dextran, MW 4 kDa, Sigma-Aldrich Japan, Tokyo, Japan) solution was added and homogenized. The subsequent process was executed using the same methods described previously to fabricate MNPs containing the model drug compounds. To puncture the MNPs, the skin samples were affixed to Parafilm. MNPs were manually punctured into the skin samples for 30 s and subsequently fixed to the skin at 12 N (1.25 kg) for 30 min. The MNPs were then removed from the skin sample and placed in the donor compartment (area 1.7 cm^2^) of a Franz diffusion cell (8mL) (CL1100-01-10, Climbing, Fukuoka, Japan) with the stratum corneum of the skin sample oriented upward. The same experimental protocol was implemented for needleless patch (NLP). The donor compartment was covered with parafilm and attached to the receptor compartment of the Franz diffusion cell. The medium used in the receptor compartment was PBS solution (PBS / pH7.4 100ml, Medicago, Quebec, Canada). A plastic container containing water at 37 ± 1 °C was positioned on a heated stirrer (RSH-1DN, AS ONE, Osaka, Japan) and agitated at 200 rpm. Samples (0.6 mL) were collected at 0, 3, 4, 5, 6, and 24 h. At each sampling, the medium in the receptor compartment was replenished with an equivalent volume of PBS solution. Each patch was tested twice (N = 2). We applied a procedure based on the experimental methods described by Surekha et al. [62] and Nagra et al. [92]. In some studies, MNPs were placed in a Franz diffusion cell while still attached to the skin; however, in this study, the MNPs were removed from the skin. The reason for this is that the results of the puncture test in this study showed that puncture marks could be observed 30 min after puncture, so we confirmed how much drug could be permeated with the shortest administration time.

After 24 h, the residual amounts of model drug compounds were examined to calculate the permeation volume. Residual amounts of MNPs on the surface of polylactic acid (MN-PLA), on the surface of skin (surface skin), and in the skin (in-skin) after puncturing the skin samples were collected using the following methods. MN-PLA and surface skin were each wiped with PBS-moistened Kimwipes and placed in 15 mL tubes, each containing 12 mL of PBS. For in-skin analysis, skin samples were sectioned to approximately 5 mm in size and placed in 15 mL tubes containing 12 mL of PBS. Tubes were extracted using a horizontal shaker (Stuart S150 orbital incubator) at 37 °C for 4 h at 200 rpm. Each sample was analyzed for fluorescence intensity at 490–520 nm using a plate reader (Infinite 200 pro, Tecan, Zurich, Switzerland).

The total amount of model drug compound in the MNP was defined as the sum of the amount of model drug compound remaining and the amount transmitted from the skin sample. The total amount of model drug compound in the MNP per area of the application site was calculated using the following formula:

Total amount = (Total model drug compound in the MNP) × (0.75 × 0.75 × π)/(MNP area)

The permeation rate per time unit was calculated using the following formula:

Permeation rate = (Permeation amount per time)/(total amount of model drug compound in the MNP per area of the application site)

## Data Availability

The datasets generated and/or analyzed during the current study are not publicly available because they belong to ongoing research projects, but are available from the corresponding author upon reasonable request.

## Supplementary Materials

The following supporting information can be downloaded at: https://www.mdpi.com/article/10.3390/gels11020089/s1, Table S1: Overview of materials used in the gas-permeable MN mold and MNP.
