# Supplementary material for: Fabrication and Evaluation of Dissolving Hyaluronic Acid Microneedle Patches for Minimally Invasive Transdermal Drug Delivery by Nanoimprinting
Source: Gels. 2025 Jan 23;11(2):89. doi: 10.3390/gels11020089 (PMC11854821; doi:10.3390/gels11020089)
Supplement: Supplementary file 1 [file gels-11-00089-s001.zip › gels-3404684-supplementary.pdf]

Table S1. Overview of materials used in the gas-permeable MN mold and MNP.

**Gas-permeable MN mold**	3-(Acryloyloxy)propyltrimethoxysilane	Gelest
	Methyltrimethoxysilane	Gelest
	Tetraethyltitanate	Gelest
	Tetraethoxysilane	Gelest
	2,4,6,8-tetramethyl-2,4,6,8-tetravinylcyclotetrasiloxane	Tokyo Chemical Industry
	2-hydroxy-2-methyl-1-phenilpropanone	Toyotsu Chemiplas
**MNP_40k**	Hyaluronic acid (30 kDa to 50 kDa)	SANCT
	Water	—
	Fluorescein isothiocyanate-dextran (4 kDa) (for permeability test)	Sigma-Aldrich Japan
**MNP_80k**	Hyaluronic acid (50 kDa to 110 kDa)	Kikkoman Biochemifa
	Water	—
	Fluorescein isothiocyanate-dextran (4 kDa) (for permeability test)	Sigma-Aldrich Japan
